# Multiple Sclerosis: Enhancing Botulinum Toxin Effects in Spasticity Management, a Systematic Review

**DOI:** 10.3390/jcm14155252

**Published:** 2025-07-24

**Authors:** Daniela Poenaru, Miruna Ioana Sandulescu, Catalin Furculescu, Claudia Gabriela Potcovaru

**Affiliations:** 1 Rehabilitation Department, Carol Davila University of Medicine, 050474 Bucharest, Romania; miruna.sandulescu@drd.umfcd.ro (M.I.S.); claudia-gabriela.potcovaru@drd.umfcd.ro (C.G.P.); 2 National Institute of Rehabilitation, 030167 Bucharest, Romania; furculescu@yahoo.com

**Keywords:** multiple sclerosis, spasticity, botulinum toxin

## Abstract

**Background/Objectives:** The objective of this review is to document the modalities to enhance the neuromuscular effects of botulinum toxin (BoNT) injection in spastic patients with multiple sclerosis (MS). **Methods:** We conducted a literature review focusing on studies involving BoNT administration for MS-related spasticity and the use of adjunctive therapies aimed at reducing dosage and increasing injection intervals. **Results:** The findings revealed a limited number of studies specific to MS patients, addressing only a few adjunct techniques, including electrical stimulation, vibration therapy, physical exercise, and extracorporeal shock wave therapy. **Conclusions:** These preliminary findings highlight the need for further research into integrative therapeutic strategies tailored specifically to the MS population.

## 1. Introduction

Multiple sclerosis (MS) is a chronic, immune-mediated, neurodegenerative, demyelinating disease that predominantly affects young adults and frequently leads to significant disability, particularly in ambulation and self-care. Spasticity and tremor—common features in MS—arise from upper motor neuron lesions and display unique characteristics in this condition [1].

Spasticity has been extensively studied across various upper motor neuron syndromes, including stroke, cerebral palsy, and MS. However, spasticity in MS exhibits distinct clinical features related to its pathophysiology, distribution, and associated symptoms. In MS, spasticity arises from demyelination and inflammation of the descending corticospinal tracts, particularly within the brainstem and spinal cord. In contrast, spasticity following stroke typically stems from focal damage to the cerebral cortex, internal capsule, or brainstem. MS-related spasticity is usually bilateral and diffuse due to widespread central nervous system involvement, whereas post-stroke spasticity is characteristically unilateral and depends on the lesion’s location. Moreover, MS spasticity has a fluctuating nature, influenced by disease activity, infections, fatigue, or changes in temperature. In contrast, stroke-induced spasticity follows a more predictable course, evolving from initial flaccidity to increased tone and, eventually, to fixed contractures. In MS, spasticity predominantly affects the lower limbs early in the disease, often with extensor dominance, resulting in a stiff gait pattern. Tremor, when present, typically involves the upper limbs. Stroke spasticity, on the other hand, presents with a flexor pattern in the upper limbs and an extensor pattern in the lower limbs, corresponding to the side of the lesion. Additionally, MS spasticity is often accompanied by painful muscle spasms (particularly in the lower limbs), fatigue, and cerebellar ataxia. In contrast, stroke-related spasticity is more commonly associated with abnormal synergistic movement patterns and pathological reflexes [2].

Botulinum neurotoxin (BoNT) is widely regarded as the gold standard treatment for spasticity management. Its mechanism of action involves the inhibition of acetylcholine release at the neuromuscular junction, leading to temporary chemodenervation and subsequent muscle relaxation. As the clinical effects of BoNT typically persist for approximately 3 to 4 months, repeated injections are required to maintain therapeutic benefit. However, long-term BoNT therapy may be associated with a reduction in efficacy due to the formation of neutralizing antibodies, a dose-dependent adverse effect. Key risk factors for antibody development include high cumulative doses and extended duration of treatment. To preserve long-term treatment effectiveness, it is essential to implement strategies that optimize BoNT administration, such as dose minimization and prolonging injection [3].

Adjunctive therapeutic interventions aimed at enhancing or prolonging the clinical effects of BoNT may play a critical role in the comprehensive management of spasticity. Optimized injection techniques, including the use of electromyographic (EMG) or ultrasound guidance, can improve targeting precision and minimize the required dose per muscle or treatment session [4]. Furthermore, several complementary modalities have been investigated for their potential to augment BoNT efficacy. A 2018 meta-analysis identified the following interventions as potentially beneficial in reducing spasticity among patients with multiple sclerosis (MS): physical exercise, electrical stimulation, vibration therapy, standing therapy, and extracorporeal shockwave therapy [5]. A subsequent meta-analysis in 2020 expanded this list to include cold therapy and electromagnetic therapy [6].

A substantial portion of research on spasticity management has concentrated on various therapeutic strategies irrespective of the underlying etiology, with a particular emphasis on post-stroke spasticity. Interventions such as electrical stimulation, vibration therapy, extracorporeal shock wave therapy, and therapeutic exercise have been extensively investigated in individuals with stroke-related spasticity.

The objective of this paper is to outline targeted strategies to enhance the efficacy of BoNT therapy in the management of spasticity in patients with MS, either by potentiating its clinical effects or minimizing the required dosage.

## 2. Materials and Methods

### 2.1. Search Strategy

We conducted a literature search across four major databases—PubMed/MEDLINE, Cochrane Library, Embase, and Web of Science—from the year 2000 to the present. The search was based on the following MeSH terms: “multiple sclerosis” AND “botulinum toxin” in combination with “physiotherapy” OR “physical exercise” OR “exercise therapy” OR “electrical stimulation” OR “vibration” OR “standing therapy” OR “shockwave therapy” OR “cold therapy” OR “electromagnetic therapy”. The manuscript was developed in accordance with PRISMA guidelines.

### 2.2. Eligibility Criteria

Inclusion criteria were randomized controlled trials, single- or double-blinded studies, non-randomized studies, pilot studies, case series, and case reports conducted on human subjects, published in English or with English abstracts. Exclusion criteria included meta-analyses, studies on animal models, and studies conducted in cell cultures.

### 2.3. Selection Process

The initial search yielded 658 titles. After removing duplicates, 53 unique titles remained. Abstracts of these studies were reviewed, resulting in the selection of eight heterogeneous studies that met our inclusion criteria.

**Figure 1:**
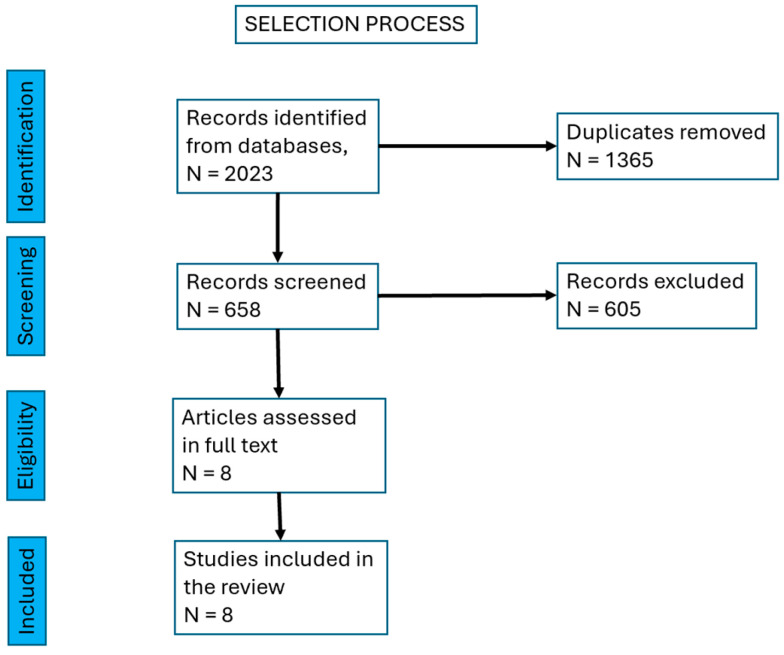
PRISMA chart showing the selection process of studies.

Figure 1, PRISMA chart for the selection process of studies.

## 3. Results

We identified two prospective, randomized, controlled, doubled blind trials [7,8], four prospective, randomized, single blind studies [9,10,11,12], one prospective non-randomized study [13], and one case report [14]. Table 1 summarizes the main features of the papers included in the analysis.

To facilitate discussion, we grouped the selected studies according to the type of therapeutic approach combined with BoNT injections.

### 3.1. Physical Exercise

A prospective, single-blind, randomized controlled pilot study conducted over a 12-week period enrolled 38 patients with multiple sclerosis (MS) presenting with focal spasticity, all of whom received BoNT-A injections. The intervention group engaged in a structured physical exercise program consisting of 15 daily sessions, each lasting 40 min. This regimen included passive and/or active exercises designed to preserve muscle length, as well as targeted stretching focused on the injected muscle groups. Assessments were conducted at 2, 4, and 12 weeks post-intervention using both objective measures—spasticity graded via the Modified Ashworth Scale (MAS)—and subjective measures—patient-reported spasticity severity using the Visual Analog Scale (VAS). The combined approach of physiotherapy and BoNT-A administration resulted in statistically significant improvements in both objective and subjective spasticity outcomes at all evaluation time points. These results underscore the therapeutic value of incorporating physical exercise into post-injection rehabilitation protocols to optimize clinical outcomes following BoNT-A treatment [9].

### 3.2. Extracorporeal Shock Wave Therapy (ESWT)

Two forms of true extracorporeal shock wave therapy (ESWT) are currently in use: focused and unfocused ESWT, while radial ESWT consists primarily of pressure waves rather than shock waves. Both focused and unfocused ESWT generate pressure waves through electromagnetic, electrohydraulic, or piezoelectric sources, characterized by very rapid pressure changes. In focused ESWT, the peak energy is concentrated deeply in the target tissue (up to 12 cm), whereas in unfocused ESWT, the waves are dispersed and penetrate to a moderate depth (4 to 6 cm). Radial ESWT is delivered via a ballistic source, producing the highest pressure at the application tip on the patient’s skin—outside the target tissue—with radial expansion and a lower penetration depth (under 3 cm). The pressure increase in radial ESWT is slower and achieves a lower peak pressure. Both focused and unfocused ESWT produce cavitation bubbles in the target tissue; the collapse of these bubbles generates secondary pressure waves. Radial ESWT produces little to no cavitation due to the slower pressure rise. The cavitation-related mechanism may play a central role in the therapeutic effects of both focused and radial ESWT [15].

#### 3.2.1. Radial ESWT

Extensive research in recent years has highlighted the spasticity-reducing effects of radial ESWT in upper motor neuron lesions. Most studies have focused on spasticity related to stroke and cerebral palsy. In MS, radial ESWT has been shown to reduce pain associated with hypertonia of the ankle extensor muscles after four weekly sessions, with the effect persisting for an additional four weeks. Although spasticity decreased after the first session, electromyographic studies did not demonstrate a reduction in spinal hyperexcitability. The presumed mechanisms of radial ESWT are likely local, acting at the level of muscle fibers, connective tissue, and the neuromuscular junction [15,16,17].

A prospective, single-blind, non-randomized clinical trial was conducted involving 16 patients with MS and spasticity, all of whom received radial extracorporeal shock wave therapy (rESWT) initiated four months following botulinum toxin (BoNT) injection into the triceps surae. The rESWT protocol consisted of four weekly treatment sessions. Outcome measures included spasticity, assessed using the Modified Ashworth Scale (MAS) and the Modified Tardieu Scale (MTS), as well as evaluations of both active and passive ankle range of motion (ROM). Assessments were performed at 30 and 90 days post-BoNT injection, and again following the final rESWT session. The results demonstrated a significant reduction in spasticity within the first 90 days post-BoNT, with a mild resurgence noted by day 120. The subsequent administration of rESWT resulted in an additional significant decrease in spasticity and a concomitant improvement in both active and passive ankle ROM, with effects sustained for at least 30 days post-treatment [13].

#### 3.2.2. Focused ESWT

Two studies investigated the effect of adding focused extracorporeal shockwave therapy (ESWT) to botulinum neurotoxin (BoNT) treatment for spasticity management.

A small trial including eight patients with MS (alongside 10 patients with stroke-related spasticity) consisted of two phases: an observational phase on BoNT administration and a prospective interventional phase involving combined BoNT injections and three weekly sessions of focused ESWT. The targeted muscles were spastic muscles of the upper limb (pectoralis major, subscapularis, elbow flexors, pronator teres, flexor pollicis longus, wrist or finger flexors) and lower limb (rectus femoris, adductors, hamstrings, triceps surae, or tibialis posterior). Outcome measures included spasticity assessed by the Ashworth Scale (AS) and gait speed measured by the 10-m walk test (10 MWT), evaluated at six months. Results showed a significant decrease in spasticity as measured by AS, longer duration of therapeutic effect, and faster gait speed in the group receiving the combined therapy [11].

Another trial involving spasticity patients, including 13 with MS, compared focused versus unfocused ESWT applied to spastic muscles of the upper limb (pectoralis major, subscapularis, elbow flexors, pronator teres, flexor pollicis longus, wrist or finger flexors) and lower limb (rectus femoris, adductors, hamstrings, triceps surae, or tibialis posterior). ESWT treatment began one week after BoNT injection and was administered once weekly for three consecutive weeks. Outcome measures were spasticity on the AS, gait speed on the 10 MWT, and patient satisfaction. The results demonstrated comparable effectiveness of both ESWT types in reducing spasticity and improving function in both the short and long term (up to six months). Regarding patient satisfaction, a higher percentage of patients reported being very satisfied in the group treated with unfocused ESWT. This may be explained by the more homogeneous wave propagation of unfocused ESWT, which potentially reaches a greater number of motor endplates and is better tolerated [12].

### 3.3. Vibration

Vibration therapy has been applied as whole-body vibration (WBV), often in the form of vibration ergometry training, as well as segmental muscle vibration.

Whole-body vibration exercise may provide a beneficial environment for rehabilitation in patients with upper motor neuron lesions. Mechanical oscillations produced during WBV induce physiological changes at multiple levels, including stimulation of skin receptors, muscle spindles, and the vestibular system, as well as alterations in cerebral activity, neurotransmitter levels, and hormone concentrations. These effects contribute to improvements in postural control, gait parameters, and coordination. WBV has been used in patients with stroke and cerebral palsy, based on evidence that vertical platform vibration elicits involuntary muscle contractions while reducing the recruitment of motor units. Since MS is associated with impaired ability to activate motor units during voluntary contraction, decreased maximal motor unit firing rates, and reduced average muscle fiber cross-sectional area, training programs involving vibration platforms have become an area of interest in this population [18].

#### 3.3.1. Whole Body Vibration

Vibration ergometry training (VET) involves exercising on an ergometer placed on a vibrating platform, thereby providing whole-body vibration. Research on the effects of vibration in patients with MS has yielded mixed results. One study found that a single 5-min session of whole-body vibration significantly improved postural stability and gait performance, with benefits evident 15 min after application and lasting up to two weeks compared to controls [19]. Conversely, a pilot study combining exercise with whole-body vibration reported only modest improvements in muscle tone and strength, potential benefits in functional mobility, and variable effects on sensation, indicating that further research is needed to clarify these outcomes [10].

A prospective pilot study was conducted in 14 patients with MS exhibiting asymmetrical lower limb involvement and moderate spasticity, to assess the impact of adding vibration ergometry training (VET) to BoNT/A treatment on gait parameters. Participants underwent five weekly sessions of whole-body vibration in conjunction with BoNT/A administration. The combination therapy did not yield significantly greater improvements in gait analysis compared to either intervention alone. The study concluded that both BoNT/A injections and VET independently contribute to gait improvement in patients with asymmetric lower limb spasticity; however, their combination did not result in additive benefits. The authors acknowledged that the findings may be constrained by the limited sample size and the relatively low vibration intensity employed, emphasizing the need for further studies to evaluate the potential synergistic effects of combined BoNT/A and VET interventions [10].

#### 3.3.2. Segmental Muscle Vibration

This technique applies a low-amplitude, high-frequency vibratory stimulus to a specific muscle using a mechanical device, inducing Ia afferent input by activating muscle spindle primary endings. The Ia inputs generated by segmental muscle vibration can modulate the excitability of the corticospinal pathway by influencing intracortical inhibitory and facilitatory circuits within the primary motor cortex.

A prospective, randomized, controlled, single-blind trial was conducted in 42 patients with MS to compare the effects of BoNT injections, segmental vibration therapy (administered as three sessions per week over four weeks), and their combination on spasticity and fatigue over a 22-week period. Spasticity was assessed using the Modified Ashworth Scale (MAS). All treatment groups demonstrated significant reductions in spasticity; however, the combination therapy produced the most sustained effects. Similarly, fatigability significantly decreased across all groups, with the combined intervention yielding the greatest improvement [8].

### 3.4. Transcranial Direct Current Stimulation

Transcranial Direct Current Stimulation (tDCS) is a non-invasive, safe, and well-tolerated brain stimulation technique that uses a low intensity (2 mA), constant electrical current delivered to the scalp to modulate neuronal activity [20].

A case report described a patient with MS presenting with upper limb intention tremor who was treated with botulinum neurotoxin (BoNT) followed by transcranial direct current stimulation (tDCS). Fifteen days after the BoNT injection, the patient underwent 15 sessions of tDCS (25 min each, five sessions per week), targeting the contralateral primary motor cortex (M1). Following BoNT administration, both functional and surface electromyography (sEMG) parameters showed improvement. These gains continued to progress with the addition of tDCS. The combined use of BoNT and tDCS appeared to yield superior clinical outcomes compared to BoNT alone [14].

### 3.5. Transcutaneous Electrical Nerve Stimulation

The effects of electrical stimulation (ES) are the enhancement of the binding and internalization, as well as the translocation, of BoNT-A at the motor nerve terminals in animal models just a few minutes after BoNT injection [21].

A prospective, randomized, double-blind trial involving five MS patients with spastic paraparesis (among other conditions) investigated the effects of two types of transcutaneous electrical stimulation following BoNT injection into the extensor digitorum brevis muscle. Participants were divided into two groups receiving either low-frequency (4 Hz) or high-frequency (25 Hz) stimulation, delivered in five daily sessions of 30 min each. Outcome measures included electromyographic (EMG) parameters—specifically, compound motor action potential (CMAP)—and clinical assessment of spasticity, evaluated on days 2, 3, 4, 10, 15, and 30. Both groups showed significant increases in CMAP amplitude, indicating enhanced neuromuscular blockade, along with reductions in spasticity. However, greater improvements were observed in the group receiving low-frequency stimulation. These findings suggest that combining BoNT with low-frequency electrical stimulation may enhance and prolong the therapeutic effects of BoNT [7].

## 4. Discussion

The present paper reviewed the existing literature to identify non-pharmacological interventions that may enhance or prolong the effectiveness of BoNT in MS patients. The selected therapies were drawn from those commonly applied in upper motor neuron-related spasticity to potentiate the effects of BoNT injections.

Physical exercise was shown to improve both functional and subjective outcomes following BoNT treatment. However, the benefits were observed only up to 12 weeks in the single available trial. These findings suggest that BoNT dosage might be reduced when combined with exercise, although no data currently supports the possibility of extending injection intervals.

Radial extracorporeal shockwave therapy administered three months after BoNT injection led to an additional 30-day reduction in spasticity, thereby extending the therapeutic effect. In contrast, focused and unfocused ESWT, when applied shortly after BoNT administration, significantly reduced spasticity and prolonged the benefits for up to six months.

The combination of vibratory ergometry training (a form of whole-body vibration therapy) with BoNT failed to demonstrate superior outcomes compared to either intervention alone. Conversely, segmental vibration applied to the injected limbs significantly reduced both spasticity and fatigue, with effects lasting up to 22 weeks. These results suggest its potential role in prolonging the interval between BoNT injections.

Electrical stimulation using low-frequency (4 Hz) currents applied to the targeted muscles resulted in greater neuromuscular blockade compared to high-frequency stimulation (25 Hz). However, outcomes were monitored only over a 30-day period, indicating possible dose reduction but offering no evidence for lengthening treatment intervals.

Finally, a novel approach targeting cortical hyperexcitability using transcranial direct current stimulation (tDCS) was reported in a case study. When combined with BoNT, tDCS contributed to further improvement in spasticity and tremor control, suggesting potential synergistic effects.

In summary, there is a limited number of studies focusing exclusively on the management of spasticity in MS; most of the existing literature involves small MS subgroups. Due to the unique characteristics of spasticity and associated phenomena in MS, it is crucial to conduct studies that specifically target MS patients.

No clinical trials to date have investigated the use of robot-assisted gait training (RAGT) or sensory integration balance training (SIBT) following BoNT administration in MS patients. However, a study published in 2014 followed 22 MS patients with Expanded Disability Status Scale (EDSS) scores ranging from 1.5 to 6.5 who participated in rehabilitation programs consisting of twelve 50-min sessions over six weeks (two sessions per week). The follow-up period lasted for one month after completion of the intervention. The results demonstrated that both RAGT and SIBT significantly improved balance, with SIBT offering additional benefits in specific balance tasks. These findings suggest that incorporating sensory integration strategies into rehabilitation programs may enhance balance and gait outcomes in individuals with MS [22].

The integration of BoNT administration with emerging rehabilitation approaches—such as RAGT, SIBT, or virtual reality—may further optimize functional recovery in MS patients and warrants future clinical investigation.

The results are systematized in Table 2.

## 5. Conclusions

There is a notable paucity of research specifically addressing therapies for MS-related spasticity and associated symptoms that aim to enhance the effectiveness of BoNT administration. As BoNT remains the gold standard for the treatment of focal spasticity, the demand for its use continues to grow in parallel with advances in MS management, which have led to improved quality of life and increased life expectancy.

Future research should focus on strategies to reduce BoNT dosage and extend the intervals between injections in order to minimize the risk of neutralizing antibody formation and to preserve long-term clinical efficacy. A multifaceted approach is essential. Accurate localization of targeted muscles through ultrasound or electromyographic guidance can reduce the required BoNT dose. Additionally, adjunctive non-pharmacologic therapeutic techniques may contribute to lowering the necessary dose and extending the duration of BoNT’s therapeutic effects.

## Figures and Tables

**Table 1:** Studies selected for the systematic review.

	Type	Intervention	Outcomes	Moments	Results	Adverse Effects
Frasson [7], 2005	Prospective, randomized, double blind, 12 pts spastic paraparesis (5 with SM)	Group I: BoNT + transcutaneous NS * (4 Hz) Group II: BoNT + transcutaneous NS (25 Hz) 5 days of 30 min/day	EMG * parameters (CMAP *), clinical assessment of spasticity	Baseline Days 2, 3, 4, 10, 15, 30	Better blockade for group I Both groups decreased spasticity, significantly better for group I	No adverse effects
Giovanelli [9], 2007	Prospective, single-blind, randomized controlled trial, 40 pts	Group I: BoNT + physical exercise (15 daily sessions, passive or active exercise and stretching) versus Group II: BoNT alone	EDSS * MAS * Self-evaluation of spasticity on a VAS	Baseline, Weeks 2, 4, 12	Significant improvement in objective and subjective spasticity at all moments Group I significantly better results	No adverse effects
Paoloni [8], 2013	Prospective, controlled, single blind, randomized, 42 pts	Group A: vibration (30 min, 120 Hz, three times a week for four weeks) Group B: vibration + BoNT Group C: BoNT Rectus femoris muscle Gastrocnemius medialis and lateralis muscles	MAS * Fatigue severity scale Barthel Index	Baseline, weeks 10 and 22	Spasticity significantly reduced in all groups, Group B longer-lasting effects, up to week 22. Fatigability improved across all groups, with a significant reduction observed in Group B.	No adverse effects
Marinaro [13], 2021	Prospective, non-randomized, single blind, controlled trial, 16 pts	BoNT, 4 months later rESWT (500 impulses, energy density flux 1.8 barr, frequency 4 Hz), 4 weekly sessions, Triceps surae muscle	MAS * MTS * ROM * active, passive	Baseline, 30 days, 90 days after BoNT injection and after rESWT administration	After BoNT, all parameters improved significantly by day 30, peaked at day 90, and then gradually declined by day 120. Following radial ESWT, all parameters showed significant improvement on day 30.	No adverse effects
Facciorusso [14], 2021	Case report, one patient	SM with upper limb tremor BoNT Week 15: transcranial direct current stimulation (tDCS)	sEMG * ADL self-questionnaire 9 HPT * BBT *	Baseline Week 15 Week 30	Functional parameters improved after BoNT and continued to improve after tDCS	Mild weakness in the extension of the fourth and fifth finger of the hand
Hefter [10], 2024	Prospective, controlled, pilot, 14 pts	Group 1: vibration ergometry training + BoNT Group 2: BoNT Leg muscles Arm muscles (according to evaluation)	Gait analysis (ground reaction forces, foot contact parameters)	Baseline Vibration protocol Control protocol	Gait parameters improved in patients with mild spasticity in both groups, no significant difference. Moderate spasticity: no functional benefit.	No adverse effects
Déniz [11], 2025	Phase 1, observational Phase 2, prospective, interventional, 8 pts (out of 18)	Phase I: BoNT alone Phase II: BoNT + fESWT, (EFD of 0.1 mJ/mm^2^, 1500 impulses at a frequency of 5 Hz) 3 weekly sessions, pectoralis major or subscapularis, the upper limb (elbow or wrist or finger flexors) and the lower limbs (rectus femoris quadriceps or triceps surae or tibialis posterior	Ashworth scale (AS) 10 MWT *	Baseline 6 months	Phase I: AS decreased with 1 point at week 5 and maintained at week 17 Phase II: AS decreased with 2 points at week 1, maintained in week 25 and significantly faster times on 10 MWT	Transient discomfort when applied in the proximity of bony surfaces, no skin reactions
Déniz [12], 2025	Prospective, randomized, controlled trial, 13 pts	BoNT + focused ESWT (3 weekly sessions) versus BoNT + unfocused ESWT (3 weekly sessions) Upper and lower limbs spastic muscles	Ashworth scale 10 MWT * Patient satisfaction	Baseline, 6 months	Both groups improved in spasticity and function (maintained from week 2 to week 26) Patient satisfaction significantly higher in unfocused group	No adverse effects

* EMG, electromyography; sEMG, surface electromyography; EDSS, Expanded Disability Status Scale; NS, nerve stimulation; CMAP, compound motor action potential; MAS, Modified Ashworth Scale; MTS, Modified Tardieu Scale; 9 HPT, 9-hole peg test; BBT, box and block test; ROM, range of motion; 10 MWT, 10-m walk test.

**Table 2:** Therapeutic approaches to enhance BoNT administration.

Therapy Added to BoNT	Moment of Application, Number of Sessions	Results/Taking into Consideration the Limited Evidence
Physical exercise	After injection, 15 daily sessions	Spasticity reduction, functional improvement
Radial ESWT	Four months after the injection, four weekly sessions	Spasticity reduction, functional improvement
Focused/unfocused ESWT	After injection, four weekly sessions	Spasticity reduction, functional improvement
Whole body vibration	After injection, five weekly sessions	Inconsistent results
Segmental body vibration	After injection, three weekly session, four weeks	Spasticity reduction, functional improvement
Transcranial direct current stimulation	After injection, five weekly sessions	Still under research
Electrical stimulation (low frequency)	After injection, five daily sessions	Spasticity reduction, functional improvement

## Data Availability

No new data were created or analyzed in this study.
